# Mage-b vaccine delivered by recombinant *Listeria monocytogenes* is highly effective against breast cancer metastases

**DOI:** 10.1038/sj.bjc.6604526

**Published:** 2008-08-19

**Authors:** S H Kim, F Castro, D Gonzalez, P C Maciag, Y Paterson, C Gravekamp

**Affiliations:** 1 California Pacific Medical Center Research Institute, 475 Brannan Street, Ste 220, San Francisco, CA 94107, USA; 2 Advaxis Inc., 675 US Highway 1, North Brunswik, NJ 08902, USA; 3 Department of Microbiology, University of Pennsylvania, 323 Johnson Pavilion, 36th St and Hamilton Walk, Philadelphia, PA 19104-6076, USA

**Keywords:** Mage-b DNA vaccine, *Listeria monocytogenes*, breast cancer metastases, 4T1 model

## Abstract

New therapies are needed that target breast cancer metastases. In previous studies, we have shown that vaccination with pcDNA3.1-Mage-b DNA vaccine is effective against breast cancer metastases. In the study presented here, we have further enhanced the efficacy of Mage-b vaccination through the improved delivery of the vaccine using recombinant *Listeria monocytogenes* (LM). Three overlapping fragments of Mage-b as well as the complete protein-encoding region of Mage-b have been expressed as a fusion protein with a truncated non-cytolytic form of listeriolysin O (LLO) in recombinant LM. These different Mage-b vaccine strains were preventively tested for their efficacy against breast cancer metastases in a syngeneic mouse tumour model 4T1. The LM-LLO-Mage-b/2nd, expressing position 311–660 of the cDNA of Mage-b, was the most effective vaccine strain against metastases in the 4T1 mouse breast tumour model. Vaccination with LM-LLO-Mage-b/2nd dramatically reduced the number of metastases by 96% compared with the saline group and by 88% compared with the vector control group (LM-LLO), and this correlated with strong Mage-b-specific CD8 T-cell responses in the spleen, after restimulation with Mage-b. However, no effect of LM-LLO-Mage-b/2nd was observed on 4T1 primary tumours, which may be the result of a complete absence of Mage-b-specific immune responses in the draining lymph nodes. Vaccination with LM-LLO-Mage-b/2nd could be an excellent follow-up after removal of the primary tumour, to eliminate metastases and residual tumour cells.

Breast cancer is the most common cancer among women around the world (Althuis *et al*, 2005), and 40% of the women diagnosed with breast cancer will progress to metastatic disease (Berkowitz *et al*, 2000). Current treatment options for localised breast cancer include surgical resection of the primary tumour, assessment of the regional lymph nodes (LNs), and removal if positive, followed by adjuvant chemotherapy or hormonal therapy (Scart *et al*, 2002). Although first-line endocrine therapy with tamoxifen or the newer third generation aromatases is promising (Kurtz and Dufour, 2002), the cure rate of metastatic breast cancer is low (Alberg and Singh, 2001). Despite aggressive treatment, for most patients the elimination of metastases or residual tumour cells after surgery is incomplete, due to chemoresistance (Pardal *et al*, 2003). Thus, metastases and not the primary tumour is the most important contributor to breast cancer morbidity and mortality. Treatments that specifically reduce or eliminate distant metastases or residual tumour cells should therefore be the focus of our efforts, and will offer the greatest promise in improving the outcome for patients with metastatic breast cancer. Enhancement of specific helper and cytotoxic T-lymphocyte (CTL) responses to breast tumours through vaccination with tumour-associated antigens (TAAs) could potentially lead to the specific elimination of micrometastases and/or residual tumour cells.

So far, many TAA have been identified in human tumours of various histological origins. The MAGE antigens are particularly interesting for the development of breast cancer vaccines, because their expression (MAGE-A and/or MAGE-B) has been frequently detected in human breast tumour biopsies (92%) (Park *et al*, 2002), but not in normal tissues (De Backer *et al*, 1995; De Plaen *et al*, 1999). Various clinical trials have shown that vaccination with MAGE-1 and -3 peptides or protein, in patients with melanoma was effective against metastases (Thurner *et al*, 1999; Marchand *et al*, 2003; Kruit *et al*, 2005; Lurquin *et al*, 2005; Van Baren *et al*, 2005). These human clinical trials not only show the potential of MAGE vaccination against metastases but also the need to further optimise the efficacy of MAGE-based vaccines to improve the clinical outcome. Such optimisations are ideally carried out in the mouse.

In previous studies, we demonstrated that DNA vaccination with mouse Mage-b in metastatic mouse breast tumour models 4TO7cg and 4T1, highly expressing Mage-b, reduced the number of metastases significantly, although not completely (Sypniewska *et al*, 2005; Gravekamp, 2007; Gravekamp *et al*, 2008). In the study presented here, we further enhanced the efficacy of Mage-b vaccine through improved delivery of the vaccine using recombinant *Listeria monocytogenes* (LM). *Listeria monocytogenes* is an intracellular pathogen that primarily infects antigen-presenting cells (APCs) such as macrophages and dendritic cells (DCs; for review see Paterson and Maciag, 2005). *Listeria monocytogenes* is an attractive vaccine vector, because proteins produced by this bacterium can be presented as short peptides through both the MHC class I and II pathways generating both CD4 and CD8 T-cell responses to these antigens (Hsieh *et al*, 1993). Direct killing of tumour cells occurs through the function of CD8 T cells, but the killing may be enhanced through the activation of CD4 T cells (Hsieh *et al*, 1993). It has been shown that vaccine antigens delivered through LM are effective against primary tumours in animal models (Pan *et al*, 1999; Gunn *et al*, 2001; Singh *et al*, 2005). As discussed earlier, metastases, and not the primary tumour, contributes most to breast cancer morbidity and mortality. In this study, we demonstrate a dramatic effect of Mage-b vaccination delivered through LM on metastases in a highly metastatic breast tumour model 4T1.

## Materials and methods

### Mice

Normal female Balb/c mice (3-month old) were obtained from Simsonsen (San Francisco, CA, USA) and maintained in the animal husbandry facility of the Pacific Medical Center Research Institute (CPMCRI) according to the Association and Accreditation of Laboratory Animal Care (AACAC) guidelines.

### Plasmids and *Listeria monocytogenes*

pcDNA3.1-Mage-b/V5 was developed in our laboratory (Sypniewska *et al*, 2005). Mouse GM-CSF plasmid (CMV1-GM-CSF) was kindly provided by Dr Stephen Johnston (the Center for Innovations in Medicine, the Biodesign Institute at Arizona State University) (Chambers and Johnston, 2003). The listerial pGG-34 plasmid, expressing the positive regulatory factor A (prfA), was developed in the laboratory of Yvonne Paterson, University of Pennsylvania, PA, USA (Singh *et al*, 2005). The prfA-negative strain XFL-7 of LM (Gunn *et al*, 2001) has been used in this study.

### Cells and cell culture

The 4T1 cell line was derived from a spontaneous mammary carcinoma in a BALB/c mouse (Aslakson and Miller, 1992). Various 4T1 sublines have been generated with different patterns of metastases (Lelekakis *et al*, 1999). The 4T1 cell line used in this study is highly aggressive, metastasizing predominantly to the mesenteric LNs (MLNs), and less frequently to the diaphragm, portal liver, spleen, and kidneys (Gravekamp *et al*, 2008). The 64pT mouse mammary tumour cell line is a spontaneous fusion between mammary cell lines 4TO7 and 68H and is non-metastatic (Rak *et al*, 1992). Both 4T1 and 64pT were kindly provided by Dr Fred Miller (Karmanos Cancer Institute, Detroit, MI, USA). Both cell lines were grown in Dulbecco's Modified Eagle's Medium (DMEM) supplemented with 10% foetal bovine serum (FBS), 1 mM mixed non-essential amino acids, 2 mM L-glutamine, insulin (0.5 HSP units per ml) penicillin (100 U ml^−1^) and streptomycin (100 *μ*g ml^−1^)

### Breast tumours and metastases

Breast tumours and metastases were generated in BALB/c mice by the injection of 10^5^ cells of the mouse mammary tumour cell line 4T1, into a mammary fat pad as described previously (Gravekamp *et al*, 2008). Primary tumours were detected by palpation within 1–2 weeks in live mice. To determine the tumour size *in situ*, the perpendicular largest diameters of the tumour were measured with a caliper. Fourteen days after injection of the tumour cell line, the mice were euthanized, weighed, and necropsied to evaluate the presence and frequency of metastases and to determine the weight and size of primary tumours. Primary tumours extended to the chest cavity lining, and predominantly metastasized to the MLNs (81%), and less frequently to the diaphragm (7%) and portal liver (4%), as well as to the surface of spleen (4%) and kidneys (4%). Metastases were visible to the naked eye as nodules. The total number of metastases per mouse (MLN, diaphragm, liver, kidney, and spleen) was determined. Normal and tumour tissues were collected aseptically and kept at −80°C, or fixed in 10% Zinc solution for 48 h and kept in 70% ethanol, until use. The primary tumours and metastases were confirmed by histology or RT–PCR. In some cases, the metastases were too small for histological analysis, and only RT–PCR for the detection of Mage-b expression (not expressed in normal cells) was performed. Normal cells do not express Mage-b.

### Construction and characterisation of LM-based Mage-b vaccine strains

Three overlapping fragments as well as the complete protein-encoding region of Mage-b were cloned in the prfA-positive plasmid pGG-34 (Gunn *et al*, 2001) under the control of a hemolysin promoter (P*hly*), and as fusion protein with a truncated non-cytolytic form of listeriolysin O (LLO). The first fragment located at the N-terminal site is 349 bp (position 3–352), the second fragment adjacent to the first fragment is also 349 bp (position 311–660), and the third fragment at the C-terminal site is 379 bp (position 610–990). The Mage-b fragments were obtained by PCR from plasmid pcDNA3.1-Mage-b/V5, generated in a previous study (Sypniewska *et al*, 2005). For each construct, a restriction site *Xho*1 (underlined) was included in the forward primer, and a myc Tag (bold), followed by a stop codon and restriction site *Xma*I (underlined) in the reverse primer. The following primers were designed to generate the first fragmant of Mage-b F^1st^/5′: CTCGAGCCTAGGGGTCAAAAGAGTAAG and R^1st^/5′:CCCGGGTTA**TAGATCTTCTTCTGAAATTAGTTTTTGTTC**A AACTTATCTAGCAGGAATTC; for the second fragment of Mage-b F2nd/5′: CTCGAGAGGAAGGCTA GTGTGCTGATA and R2nd/5′: CCCGGGTTA**TAGATCTTCTTCTG AAATTAGTTTTTGTTC**T CCATGCAGAAATTGCCAGAC were designed; and for the third fragment of Mage-b F^3rd^/5′: CTCGAGAACCGTGCCACTGAGCAAGAG and R^3rd^/5′: CCCGGG**TTATAGATCTTCTTCTGAAATTAGTTTTTGTTC**C ATGTTAGAGGACTTTTGGGA were designed. The Mage-b fragments or complete Mage-b were cloned into the listerial pGG-34 plasmid by digestion of the PCR products of Mage-b as well as the pGG-34 plasmid with *XHo*I and *Sma*I, followed by purification of the digests and ligation of pGG-34 with MAge using T4 DNA polymerase (Invitrogen, Life Technologies) and transformed into *Escherichia coli*. Positive colonies were analysed by restriction digestion with *XHo*I and *Sma*I, and DNA sequencing. Subsequently, the plasmids of positive colonies were transformed into the recombinant prFA-negative LM strain XFL-7 (Hsieh *et al*, 1993) and analysed for the secretion of the MAge proteins by western blotting as described below. The LM-LLO used in this study is attenuated, that is, the coding region for the C-terminal part of the LLO (cytolytic domain that binds cholesterol in the membranes) protein has been deleted, and mutations have been introduced into the prfA gene (expressed by the pGG34 vector), which reduced the pathogenicity of the LM (Singh *et al*, 2005).

### Western blotting

The Mage-b LM-based vaccines were grown overnight in Luria–Bertani medium with 50 *μ*g ml^−1^ of chloroamphenicol at 37°C. Supernatants were precipitated with trichloro acetic acid (TCA) and resuspended in SDS sample buffer (Invitrogen, Life Technologies). Twenty microliters of each sample was loaded on a 4–12% Bis-Tris SDS–PAGE gel (Invitrogen, Life Technologies). The proteins were then transferred to a nitrocellulose membrane and probed with a rabbit polyclonal antiserum raised to the first 30 residues of the LLO protein (anti-proline, glutamic acid, serine, and threonine (PEST) (Pan *et al*, 1999). The secondary antibody (Ab) was an HRP-conjugated anti-rabbit Ab (Pharmingen). In addition, transferred proteins were probed with mouse anti-myc Abs, followed by a goat antimouse IgG conjugated with HRP as secondary Ab.

### RT–PCR and southern blotting

RNA was isolated using Trizol according to the manufacturer's instructions (Life Technologies, Carlsbad, CA, USA). Conversion of 1 *μ*g of mRNA into cDNA was performed with Superscript Preamplification system (Life Technologies). Subsequently, 10 *μ*l of the cDNA was amplified by hot start PCR (Platinum PCR SuperMix, Life Technologies; 40 cycles at 94°C for 30 s, 58°C for 30 s, 72°C for 2 min) in a thermocycler from Perkin-Elmer (Norwalk, CT, USA). To detect the expression of all three genes, that is, *Mage-b1*, *-b2*, or *-b3*, we used the set of primers F111 5′-GAGCTTGATCCACGAGTTC-3′and R769: 5′-AGGAGACCTGTCCTAGGC-3′ published by De Backer *et al* (1995). The forward primer is located in the second exon and the reverse primer in the third exon of *Mage-b2*, amplifying a 658 bp fragment*. β-*actin was used as an internal control for RNA quality. Primers for *β-*actin were 5′-TCATGAAGTGTGACGTTGACATCCGT-3′, and 5′-CCTAGAAGCATTTGCGGTGCACGATG-3′ (Life Technologies). RT–PCR products were separated in an ethidium–bromide-stained agarose gel, and transferred to an immobilon-N^+^ membrane (Amersham, Buckinghamshire, England) and hybridised with a chemiluminescence-labelled and sequenced 993 bp MAge-specific probe (AY196960) according to the manufacturer's instructions (enhanced chemiluminescence; Amersham).

### Immunisation and tumour challenge

The LD_50_ of each Mage-b vaccine strain was determined by vaccinating five mice with various doses of each vaccine strain (10^5^, 10^6^, 10^7^ 10^8^ colony-forming units (CFU)) in 500 *μ*l saline. The LD_50_ for all constructs was 10^8^ CFU.

**Figure 1:**
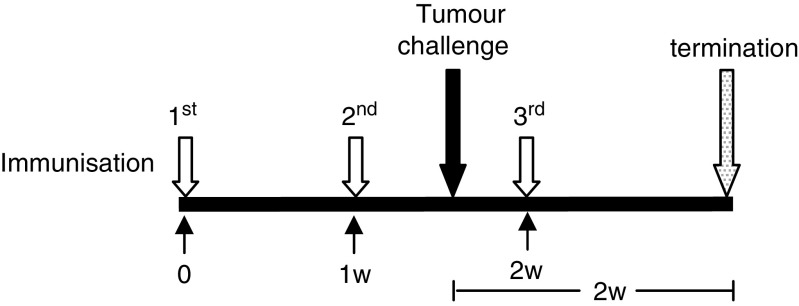
Schematic view of immunisations and tumour challenge. BALB/c mice were immunised three times intraperitoneally with 0.1 × LD_50_ of each Listeria-based Mage-b vaccine strain or with the vector control strain LM-LLO, or saline, with 1-week time intervals. Four days after the second immunization, mice were injected with 10^5^ 4T1 tumour cells in a mammary fat pad. Two weeks after tumour challenge, mice were euthanized and analysed.

To determine the efficacy of each Mage-b vaccine strain, Balb/C mice were immunised intraperitoneally (three times; 1 week time interval) with 0.1 × LD_50_ of each construct separately or combined (LM-LLO-Mage-b/1st, LM-LLO-Mage-b/2nd, LM-LLO-Mage-b/3d, LM-LLO-Mage-b/complete), or with 0.1 × LD_50_ of the control vector (LM-LLO), or with saline. For tumour induction, mice were injected with 10^5^ 4T1 tumour cells into a mammary fat pad 4 days after the second immunisation. Fourteen days after tumour challenge, the mice were euthanized and analysed for tumour size, frequency, and location of metastases. A schematic view of the immunisations and tumour challenge is given in Figure 1.

### *In vitro* analysis of Mage-b-specific immune responses

Cells from draining (inguinal) LNs and spleens were isolated according to standard protocols (Reeves and Reeves, 2003) from BALB/c mice with or without 4T1 tumours, that were immunised three times with 0.1 × LD_50_ of the vaccine (LM-LLO-Mage-b/2nd), or with 0.1 × LD_50_ the control vector (LM-LLO), or saline. Within each group, the spleen cells were pooled. Briefly, 2 × 10^5^ cells from spleens or LNs were restimulated with 5 × 10^4^ bone marrow (BM) cells (transfected with pcDNA3.1-Mage-b plasmid DNA and pCMV-GM-CSF plasmid DNA (1 *μ*g of each plasmid DNA per 5 × 10^6^ BM cells), using the Nucleofector kit of AMAXA (Gaithersburg, MD, USA), and cultured in 200 *μ*l of RPMI containing 10% FBS. Two days later, the frequency of IFN*γ*- and interleukin (IL)-2-producing cells was determined by ELISPOT according to the standard protocols (Pharmingen, San Diego, CA, USA). Positive cells were counted by an ELISPOT reader (CTL Immunospot S4 analyzer, Cellular Technology Ltd, Cleveland, OH, USA). Spleen cells were depleted for CD8 T cells, using magnetic bead depletion techniques according to the manufacturer's instructions (Miltenyi Biotec Inc., Auburn, CA, USA). Fluorescence-activated cell sorting analysis showed that ⩾90% of all CD8 T cells were removed after depletion (data not shown).

### The effect of IL-6 on Mage-b-specific immune responses

To test the effect of IL-6 on Mage-b-specific immune responses *in vitro*, purified IL-6 (100 pg ml^−1^) was added or not to the wells with spleen cells (2 × 10^5^) and BM cells expressing Mage-b (5 × 10^4^) in 200 *μ*l of RPMI containing 10% FBS. Two days later, the number of IFN*γ*-producing cells was determined by ELISPOT reader as described above. In addition, anti-IL-6 antibodies (50 *μ*g ml^−1^) were either or not added to the wells with LNs (2 × 10^5^) and syngeneic 64pT tumour cells (1 × 10^4^; treated with mitomycin C; expressing highly Mage-b; Gravekamp *et al*, 2004), and secreting highly IL-6 (3000 pg ml^−1^) (Gravekamp *et al*, 2008), in 200 *μ*l of RPMI containing 10% FBS. Two days later, the production of IFN*γ* was determined by quantitative ELISA as described previously (Sypniewska *et al*, 2005). Anti-IL-6 antibodies and purified IL-6 were purchased from Pharmingen.

## Results

### Generation of the LM strains that secrete Mage-b

**Figure 2:**
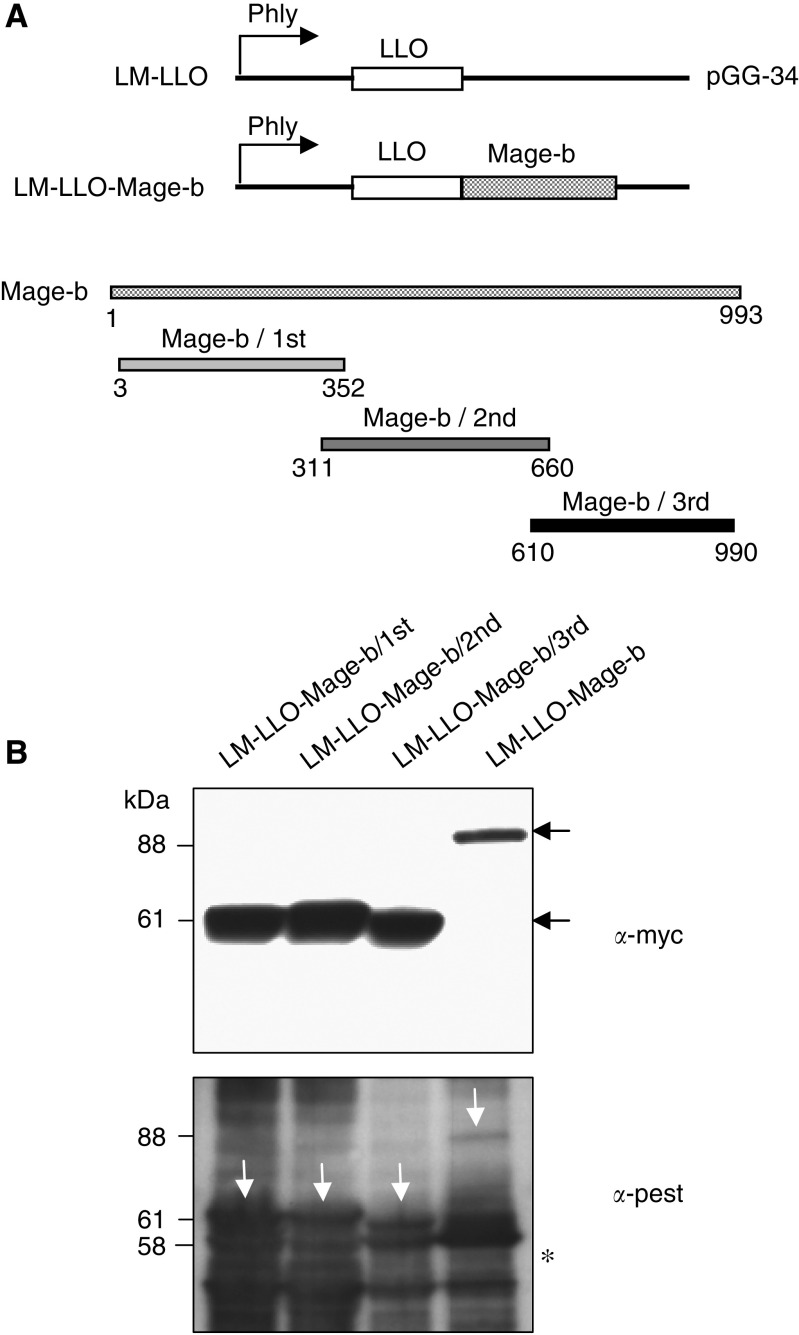
Construction and characterisation of Listeria-based Mage-b vaccine strains. (**A**) Three overlapping fragments of mouse Mage-b (homologous to human MAGE-B) were cloned as a fusion protein with a truncated non-cytolytic listeriolysin O (LLO) in the pGG-34 vector under the control of the listerial hemolysin promoter (P*hly*). (**B**) Secretion of LLO-Mage-b proteins by the Listeria-based vaccine strains was detected by western blotting using *α*-myc antibodies (top) and *α*-pest antibodies (bottom). In the western blot with *α*-myc antibodies, the LLO-Mage-b proteins are indicated by black arrows, and in the western blot with *α*-pest antibodies by white arrows. The complete Mage-b protein fused with truncated LLO represents a band of 88 kDa, whereas the three fragments of Mage-b fused with truncated LLO represent a band of 61 kDa. Endogenous LLO (58 KDa) secreted by all LM is indicated by a star.

Three recombinant LM strains that express and secrete overlapping fragments of Mage-b, as well as the complete protein-encoding region of Mage-b have been designed and constructed (Figure 2A). These fragments of Mage-b were selected as alternative antigens for the following reasons: (1) to lose function of Mage-b (complete Mage-b may induce the growth of tumour cells (Yang *et al*, 2007), (2) smaller fragments are easier to secrete than larger fragments, and (3) to select the fragment with the most protective effect *in vivo*. All fragments have been cloned into the listerial pGG-34 expression vector as fusion protein with LLO. Listeriolysin O was used for its ability to improve the immunogenicity of poor immunogenic self-antigens (Singh *et al*, 2005). The secretion of Mage-b protein of each fragment, as well as the complete protein-encoding region of Mage-b has been confirmed by western blotting of the secreted listerial proteins (Figure 2B). The secreted fusion proteins LM-LLO/Mage-b/1st, 2nd, and 3rd are 61 kDa, whereas the LM-LLO-Mage-b/complete is 88 kDA. It is clear from Figure 2B that the Mage-b fragments were more efficiently secreted than the complete Mage-b.

### Mage-b transcripts in 4T1 primary tumours and metastases

**Figure 3:**
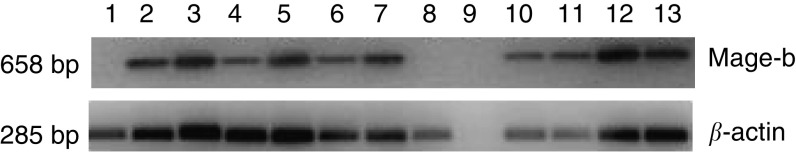
Expression of Mage-b in 4T1 primary tumours and metastases. The Mage-b-specific RT–PCR product of 632 bp was detected by southern blotting using DNA probe encoding Mage-b. *β*-actin (285 bp) was used to determine RNA quality. The lanes were loaded as follows: lane 1: normal breast tissue; lane 2: 4T1 tumour; lane 3: 4T1 tumour; lane 4: metastasis in peritoneal cavity (PC); lane 5: metastasis in PC; lane 6: metastasis liver; lane 7: metastasis liver; lane 8: metastasis spleen; lane 9: metastasis spleen; lane 10: metastasis kidney; lane 11: metastasis kidney; lane 12: metastasis diaphragm; lane 13: metastasis diaphragm.

To confirm the expression of Mage-b in the 4T1 primary tumours and metastases (MLNs, diaphragm, portal liver, spleen, and kidneys), we randomly analysed tissue samples from primary tumours and metastases by RT–PCR and southern blotting (Figure 3). All primary tumours and metastases, except two metastases present on the surface of the spleen, did express Mage-b. From one metastasis, the quality of the mRNA was poor, as indicated by the absence of *β*-actin transcription products. The other metastasis may have lost Mage-b expression.

### Preventive effect of vaccination with the various LM-based Mage-b vaccine strains

**Figure 4:**
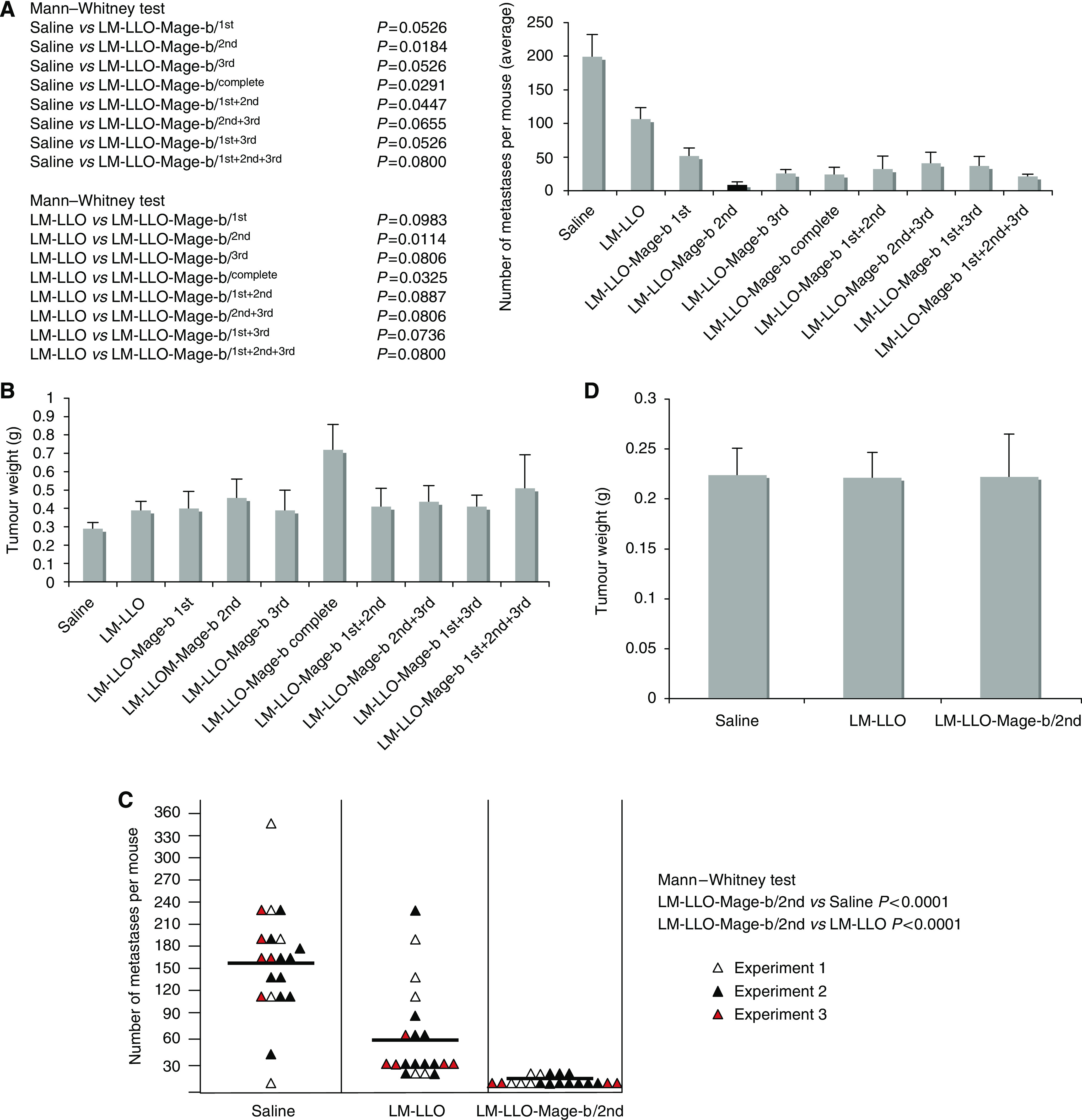
Strong effect of vaccination with LM-LLO-Mage-b/2nd on metastases but not on primary tumours in the 4T1 model. BALB/c mice were immunised with the various Listeria-based Mage-b vaccine strains and challenged with 4T1 tumour cells as outlined in Figure 1. Two weeks after tumour challenge, mice were euthanized and the number of metastases (**A**) and tumour size (**B**) was determined per mouse. LM-LLO-Mage-b/2nd was the most effective vaccine strain against 4T1 metastases, whereas none of the Listeria-based Mage-b vaccines had any inhibitory effect on tumour growth. These vaccine studies were repeated three times in independent experiments with the most effective vaccine strain, that is, the LM-LLO-Mage-b/2nd. Again, the number of metastases (**C**) and tumour size (**D**) was determined per mouse. Results were averaged and subjected to statistical analysis using Mann–Whitney test (*n*=5 mice per group in each experiment). The error bars represent the s.e.m. In panel C, each triangle represents the number of metastases per mouse, and the vertical bars represent the average number of metastases per mouse.

The preventive effect of each LM-based Mage-b vaccine strain on metastases and primary tumours has been determined in the 4T1 model. Mice were immunised with the vaccine strains and challenged with 4T1 tumour cells as outlined in Figure 1. It appeared that LM-LLO-Mage-b/2nd was the most effective vaccine strain (Figure 4A). LM-LLO-Mage-b/2nd vaccination significantly reduced the number of metastases by 96% compared with the saline group and by 92% compared with the vector control group (LM-LLO). The average number of metastases as determined for each group was 9 (LM-LLO-Mage-b/2nd), 104 (LM-LLO), and 199 (saline). However, none of the Mage-b vaccine strains reduced the growth of the primary tumours (Figure 4B). Moreover, the LM strain expressing the complete Mage-b enhanced the tumour growth by more than two times compared with the saline group (Mann—Whitney, *P*=0.0005). Therefore, LM-LLO-Mage-b complete was excluded from further vaccine studies. In previous studies, we have shown that complete Mage-b is able to enhance tumour growth (Yang *et al*, 2007). Our results suggest that LM-LLO-Mage-b may have a direct effect on the tumour cells. This idea is currently under investigation.

In summary, LM-LLO-Mage-b/2nd was the most effective strain and therefore selected for further analysis. Vaccine studies with LM-LLO-Mage-b/2nd were repeated to confirm its dramatic effect on the metastases, and to analyse Mage-b-specific immune responses *in vitro*.

Vaccinations with LM-LLO-Mage-b/2nd were repeated three times in the 4T1 model. Immunization with LM-LLO-Mage-b/2nd significantly reduced the number of metastases by 96% compared with the saline group and by 88% compared with the vector control group (LM-LLO; Figure 4C). The average number of metastases per group as determined was 7 (LM-LLO-Mage-b/2nd), 59 (LM-LLO), and 158 (saline). Again, while the effect was dramatic on the metastases, no effect of LM-LLO-Mage-b/2nd was observed on the primary tumour (Figure 4D).

Pure preventive immunizations, that is, three immunisations followed by tumour challenge, 10 days after the last immunisation, resulted also in a significant reduction in the number of metastases in the mice immunised with LM-LLO-Mage-b/2nd compared with the saline group (Mann—Whitney, *P*=0.0159) but not compared with the control vector group (data not shown). Moreover, two immunisations before and one after tumour challenge were more effective than three preventive immunisations.

### Mage-b-specific immune responses *in vitro*

**Figure 5:**
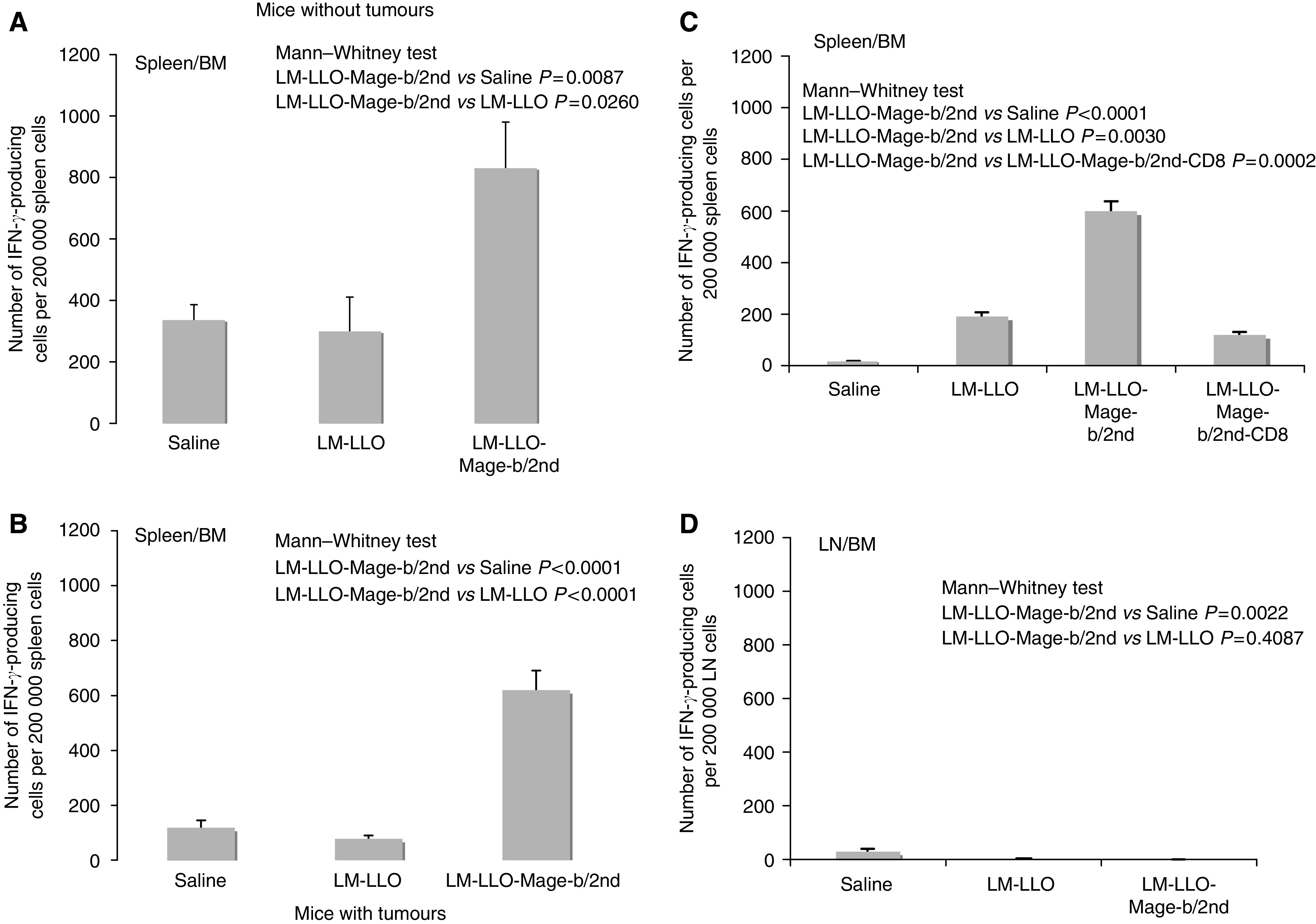
Mage-b-specific immune responses *in vitro*. BALB/c mice were immunised with the LM-LLO-Mage-b/2nd vaccine strain and challenged with 4T1 tumour cells as outlined in Figure 1, or not challenged with 4T1 tumour cells. Two weeks after tumour challenge, mice were euthanized and spleens and draining (inguinal) lymph nodes (LNs) were analysed for *in vitro* immune responses upon restimulation with Mage-b. For this purpose, the number of IFN*γ*-producing cells in spleens of mice without (**A**) and with (**B**) 4T1 tumours and metastases were compared. Again, the number of IFN*γ*-producing cells were determined in spleens (**C**) but now compared with the number of IFN*γ*-producing cells in the LNs (**D**) of mice bearing 4T1 tumours and metastases were compared. Lymph nodes and spleens were from the same mice, and tested in the same experiment. In panel C, spleen cells depleted for CD8 T cells are shown as well. All restimulation assays were performed with bone marrow (BM) cells transfected with pcDNA3.1-Mage-b and pCMV1-GM-CSF plasmid DNA. Two days later, Mage-b-specific immune responses were analysed by ELISPOT. Controls such as BM cells transfected with pcDNA3.1-Mage-b, or with pCMV-GM-CSF, or non-transfected BM cells did not produce IFN (data not shown). The LM-LLO-Mage-b/2nd vaccine was tested in three independent experiments. Results were averaged and subjected to statistical analysis using Mann–Whitney test (*n*=5 mice per group in each experiment). The error bars represent the s.e.m.

Mage-b-specific immune responses were analysed in spleen and LNs of vaccinated and control mice. First, vaccinated and control mice without 4T1 tumours and metastases were analysed for Mage-b-specific immune responses. A significant increase was observed in the number of IFN*γ*-producing cells in the group of LM-LLO-Mage-b/2nd compared with the control groups, demonstrating the high immunogenicity of Mage-b/2nd (Figure 5A). Second, vaccinated and control mice bearing 4T1 tumours and metastases were analysed for Mage-b-specific immune responses. A significant increase in the number of IFN*γ*-producing cells was observed in the group of LM-LLO-Mage-b/2nd compared with the control groups (Figure 5B), demonstrating that LM-LLO-Mage-b/2nd was able to induce Mage-b-specific immune responses even in mice with 4T1 tumours. Finally, immune responses in the spleens were compared with immune responses in the draining LNs (at the site of the primary tumours), both isolated from the same mice with 4T1 tumours and metastases, and restimulated in the same experiment, as described above. While strong Mage-b-specific immune responses were observed in the spleen (Figure 5C), those immune responses were completely absent in the draining LNs (Figure 5D). Depletion of CD8 T cells showed a decrease in the number of IFN*γ*-producing cells in the spleen by 80% (Figure 5C). No significant increase was observed in the number of IL-2-producing cells of the group of vaccinated mice compared with the control mice and therefore not shown.

### The effect of IL-6 on Mage-b-specific immune responses *in vitro*

**Figure 6:**
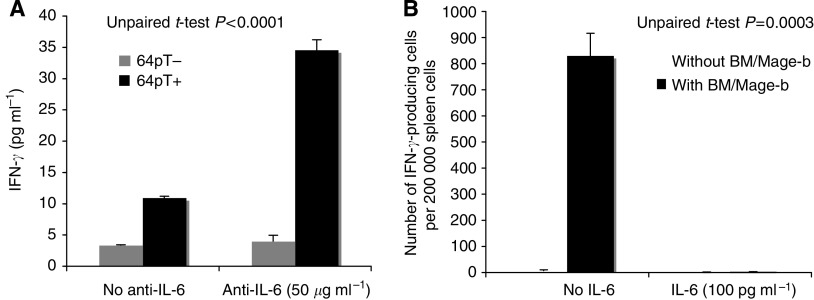
Effect of IL-6 on Mage-b-induced immune responses *in vitro*. To analyse the effect of IL-6 on Mage-b-specific immune responses *in vitro*, draining lymph nodes (LNs) of 4T1 tumour-bearing mice were cocultured with or without 64pT tumour cells, expressing highly Mage-b, and producing high levels of IL-6. These cocultures were performed in the absence or presence of anti-IL-6 antibodies (**A**). After 2 days of stimulation, the production of IFN*γ* was determined with quantitative ELISA. In this experiment, the lymph nodes (LNs) of 10 mice were pooled. In addition, spleen cells of 4T1 tumour-bearing mice were cocultured with or without autologous bone marrow (BM) cells transfected with pcDNA3.1-Mage-b and pCMV-GM-CSF. These cocultures were performed in the absence or presence of purified IL-6 (**B**). After 2 days of stimulation, the number of IFN*γ*-producing cells was determined with an ELISPOT reader. This experiment was performed twice with spleens of mice that received vaccination with LM-LLO-Mage-b/2nd. In each experiment, spleens of five mice were pooled. Controls such as BM cells transfected with pcDNA3.1-Mage-b and/or pCMV-GM-CSF, or non-transfected BM cells, did not produce IFN*γ* (data not shown). The results of both assays were subjected to statistical analysis using the unpaired *t*-test. The error bars represent the s.e.m.

As shown in Figure 5C and D, Mage-b-specific CD8 T-cell responses were present in the spleen, but completely absent at the site of the primary tumours (in draining LN). This implies that at the site of the primary tumours, either Mage-b-specific CD8 T cells are absent, or that Mage-b-specific CD8 T cells are present but failed to function, for example, by the factor(s) produced by the primary tumours. We analysed this latter possibility. In previous studies, we found that 4T1 primary tumours produced high levels of IL-6 (Gravekamp *et al*, 2008). Interleukin-6 is a potential candidate for T-cell inhibition. In the current study, the effect of anti-IL-6 antibodies as well as of purified IL-6 on Mage-b-induced immune responses has been analysed *in vitro*. For this purpose, anti-IL-6 antibodies were added to LNs of 4T1 tumour-bearing mice, restimulated with Mage-b. To avoid the generation of immune responses against all other TAA than Mage-b, we used 64pT instead 4T1 tumour cells in the restimulation assay. 64pT is a syngeneic breast tumour cell, highly expressing Mage-b (Gravekamp *et al*, 2004) and highly secreting IL-6 (3000 pg ml^−1^) (Gravekamp *et al*, 2008). Indeed, addition of anti-IL-6 antibodies to the *in vitro* restimulation assay significantly increased the production of IFN*γ*, whereas the production of IFN*γ* could not be induced in the same restimulation assay without anti-IL-6 antibodies (Figure 6A). In accordance with this result, the addition of purified IL-6 to spleen cells of 4T1 tumour-bearing mice that were immunised with LM-LLO-Mage-b/2nd, completely inhibited the generation of IFN*γ*-producing cells upon restimulation with BM cells highly expressing Mage-b, while in the absence of purified IL-6, a high number of IFN*γ*-producing cells was detected (Figure 6B).

## Discussion

Clinical trials have shown that vaccination with MAGE has effect on metastases (Thurner *et al*, 1999; Marchand *et al*, 2003; Kruit *et al*, 2005; Lurquin *et al*, 2005; Van Baren *et al*, 2005), but improvement of MAGE vaccines is strongly needed. In previous studies, we have shown that immunisation with Mage-b combined with GM-CSF plasmid DNA and thioglycollate reduced the number of metastases by 65% compared with the control group in a highly metastatic breast tumour model, 4T1 (Gravekamp *et al*, 2008). In this study, we have further improved Mage-b vaccination by using an improved delivery system, that is, recombinant LM. *Listeria monocytogenes* infects primarily APC such as macrophages and DCs, and delivers the Mage-b antigen with high efficiency to the APC. Three overlapping fragments of Mage-b (LM-LLO-Mage-b/1st, LM-LLO-Mage-b/2nd, and LM-LLO-Mage-b/3rd) as well as the complete protein-encoding region of Mage-b (LM-LLO-Mage-b/complete) have been expressed in recombinant LM. Each fragment of Mage-b as well as the complete Mage-b is secreted as a fusion protein with a truncated, non-cytolytic form of LLO. Most effective was the LM-LLO-Mage-b/2nd vaccine strain. Vaccination with LM-LLO-Mage-b/2nd dramatically reduced the number of metastases by 96% compared with the saline group and by 88% compared with the vector control group, and this correlated with strong Mage-b-specific CD8 T-cell responses in the spleen upon restimulation with Mage-b. These results suggest that LM-LLO-Mage-b/2nd (position 311–660 of the cDNA of Mage-b) may contain a higher number of, or more effective protective epitope(s) than LM-LLO-Mage-b/1st or LM-LLO-Mage-b/3rd. No further analysis has been performed in this study to identify protective epitope(s) within the Mage-b protein. We have studied Mage-b-specific CD8 T cells secreting IFN*γ*, as tumour cell kill is mediated through CD8 function (Singh *et al*, 2005). However, recent studies show that CD4 helper T cells, secreting IFN*γ*, may play an important role in tumour rejection as well (Qian *et al*, 2004; Chikamatsu *et al*, 2008).

The LM-LLO had an effect on the metastases, and it significantly reduced the number of metastases compared with the saline group. Evidence exists that LM-LLO itself can activate CD8 T cells, as well as NK cells and NK DCs (NKDCs) to produce IFN*γ in vivo* (Messingham *et al*, 2007; Plitas *et al*, 2007). Indeed, we found IFN*γ*-producing CD8 T cells, NK cells, and macrophages, in spleen cultures of mice immunised with LM-LLO or LM-LLO-Mage-b/2nd, restimulated with LM-LLO (data not shown). IFNγ has an antimetastatic effect (Hayakawa *et al*, 2002), and may explain the effect of LM-LLO on the metastases.

Despite the dramatic effect on the metastases, no reduction in the growth of 4T1 primary tumours was observed in mice vaccinated with the LM-LLO-Mage-b/2nd or with the other MAge vaccine strains compared with the control groups. In correlation with this effect, we found that Mage-b-specific immune responses were completely absent in the LNs at the site of the primary tumours. However, both 4T1 tumours and metastases expressed high levels of Mage-b. This implies that Mage-b-specific CD8 T cells are either absent, or present but inhibited in function at the site of the primary tumours. In previous studies with pcDNA3.1-Mage-b vaccination, we found that the number of CD8 T cells significantly increased in the 4T1 tumours of Mage-b vaccinated mice compared with the control groups, despite very poor Mage-b-specific immune responses (IFN*γ* production) in the draining LNs (Gravekamp *et al*, 2008). With this in mind, the inhibition of T-cell function may be more likely than the absence of Mage-b-specific CD8 T cells at the site of the primary tumours. Many tumours produce and accumulate lymphokines or factors at high levels that may inhibit vaccine-induced T-cell responses such as IL-6, transforming growth factor (TGF*β*), IL-10, cyclooxygenase (COX)-2, and its product prostaglandine E, PD1-ligand or indolamine, 2,3-dioxygenase (IDO; Gajewski *et al*, 2006). In a previous study, we found that IL-6 and TGF*β* are highly produced by the 4T1 primary tumours and metastases (Gravekamp *et al*, 2008). Evidence exists that TGF*β* may induce regulatory T cells (T_regs_) (DiPaolo *et al*, 2007) at the site of the primary tumour. Indeed, very recently, it has been reported that 4T1 tumours are massively infiltrated with T_regs_ preventing efficient activation of CD8 T cells (Chaput *et al*, 2007). In humans also T_regs_ cells have been found at the site of the primary tumours, including breast cancer (Liyanage *et al*, 2002).

In addition to TGF*β*, we found suggestive evidence that IL-6 may have contributed to T-cell unresponsiveness as well. Interleukin-6 is a potent regulator of DC differentiation (Park *et al*, 2004), and is able to initiate the expression of STAT3 in DC. High levels of STAT3 can prevent the maturation of DC and subsequent presentation of antigens (Xie *et al*, 2003), resulting in T-cell inhibition. Both 4T1 primary tumours and metastases secrete IL-6 (Gravekamp *et al*, 2008). However, the primary tumours are 100–100 000 times larger than the metastases. Therefore, it is expected that the accumulation of IL-6 is much higher in the environment of the primary tumours than in the environment of the metastases. We then hypothesised that the high accumulation of IL-6 may have downregulated Mage-b-specific immune responses at the site of the primary tumour. To evaluate this hypothesis, we analysed the effect of IL-6 on Mage-b-specific immune responses *in vitro*. Indeed, IL-6-neutralising antibodies were able to restore Mage-b-specific immune responses in draining LNs of 4T1 tumour-bearing mice *in vitro* when restimulated with Mage-b, whereas purified IL-6 completely prevented the induction of Mage-b-specific immune responses in the spleen of 4T1 tumour-bearing mice, when restimulated with Mage-b. Here, we demonstrated that IL-6 contributed to the inhibition of Mage-b-specific immune responses *in vitro*. Whether IL-6 has contributed to T-cell inhibition at the site of the primary tumour *in vivo* needs to be further analysed. High levels of IL-6 have been found in many human breast cancers (Kuang *et al*, 1998; Sotiriou *et al*, 2001). Moreover, IL-6 promotes tumour growth (Celis *et al*, 2005) and may induce chemoresistance (Conze *et al*, 2001). Therefore, agents that inhibit the production of IL-6 could improve the efficacy of vaccination or chemotherapy against breast cancer.

Finally, we have studied safety issues of the LM-LLO-Mage-b/2nd vaccine. It is known that LM infects kupfer cells and hepatocytes in the liver, macrophages in the spleen, and epithelial cells in the gastro intestines (GI) (Vazquez-Boland *et al*, 2001). However, the attenuated Listeria bacteria will be cleared by the immune system within 3–5 days after immunisation. After three immunisations with LM-LLO-Mage-b/2nd, we found some inflammatory spots in the liver, but not in the spleen or GI. Moreover, LM-LLO-based vaccines have been already tested in cancer patients in phase I/II clinical trials, and flu-like symptoms are the only side effects observed (Rothman, 2008).

In summary, we have demonstrated here that vaccination with LM-LLO-Mage-b/2nd reduced the number of 4T1 metastases dramatically in correlation with robust Mage-b-specific CD8 T-cell responses *in vitro*. Unfortunately, no effect was observed on the primary tumours, and this was correlated with the complete absence of Mage-b-specific T-cell responses *in vitro*. Therefore, our results suggest that vaccination with MAGE-B/2nd may dramatically improve the clinical outcome of breast cancer therapy, if applied after removal of the primary tumour.
